# Supplementary material for: Immunogenicity and efficacy of vaccine boosters against SARS-CoV-2 Omicron subvariant BA.5 in male Syrian hamsters
Source: Nat Commun. 2023 Jul 17;14:4260. doi: 10.1038/s41467-023-40033-2 (PMC10352277; doi:10.1038/s41467-023-40033-2)
Supplement: Supplementary file 1 — Supplementary Information [file 41467_2023_40033_MOESM1_ESM.pdf]

Supplementary Information

Immunogenicity and efficacy of vaccine boosters against SARS-CoV-2 Omicron subvariant BA.5 in male Syrian hamsters

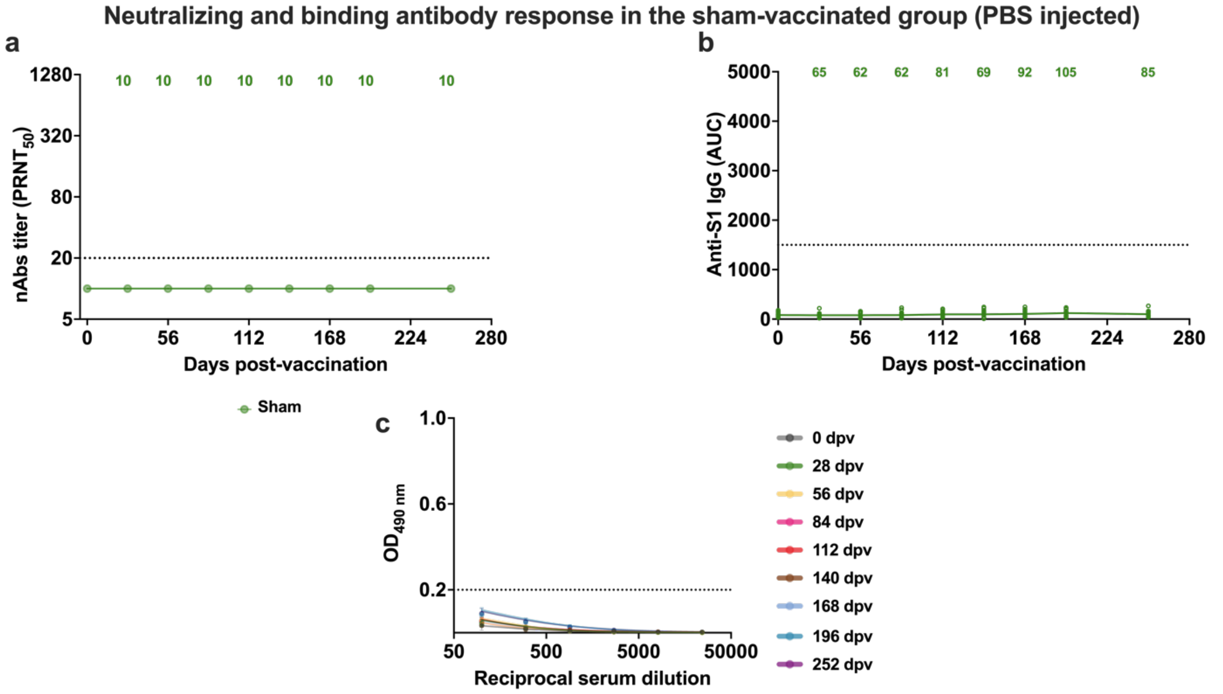

Supplementary Fig. 1. Kinetics of neutralizing and binding antibody response against WA1/2020 in sham vaccinated hamsters (PBS injected). a Serum neutralizing antibody responses up to 252 days after PBS injection as judged by plaque reduction neutralization test (PRNT) based on 50% or greater reduction in plaque counts ($PRNT_{50}$) against WA1/2020 strain in the immunized hamsters (n=15/per group). **b** WA1/20 anti-S1 IgG normalized area under the curve (AUC) kinetics as judged by ELISA. GMT values are indicated at the top of the graphs. The fitted curve lines are created by Fit Spline program of GraphPad Prism 9.4 software where shadows indicating 95% confidence intervals, symbols illustrate geometric mean titer (GMT) values, dotted lines show the LOD. **c** Serum reactivity to WA1/20 spike-S1 protein up to 252 dpv. Optical density at 490 nm ($OD_{490\ nm}$) for the indicated reciprocal dilutions. Dots and error bars represent GMT and 95% CI, respectively. Sigmoidal 4-Parameter Logistic (4PL) regression curve fitting was used to plot all ELISA data, using GraphPad Prism 9.4 software. See Source Data for complete data.

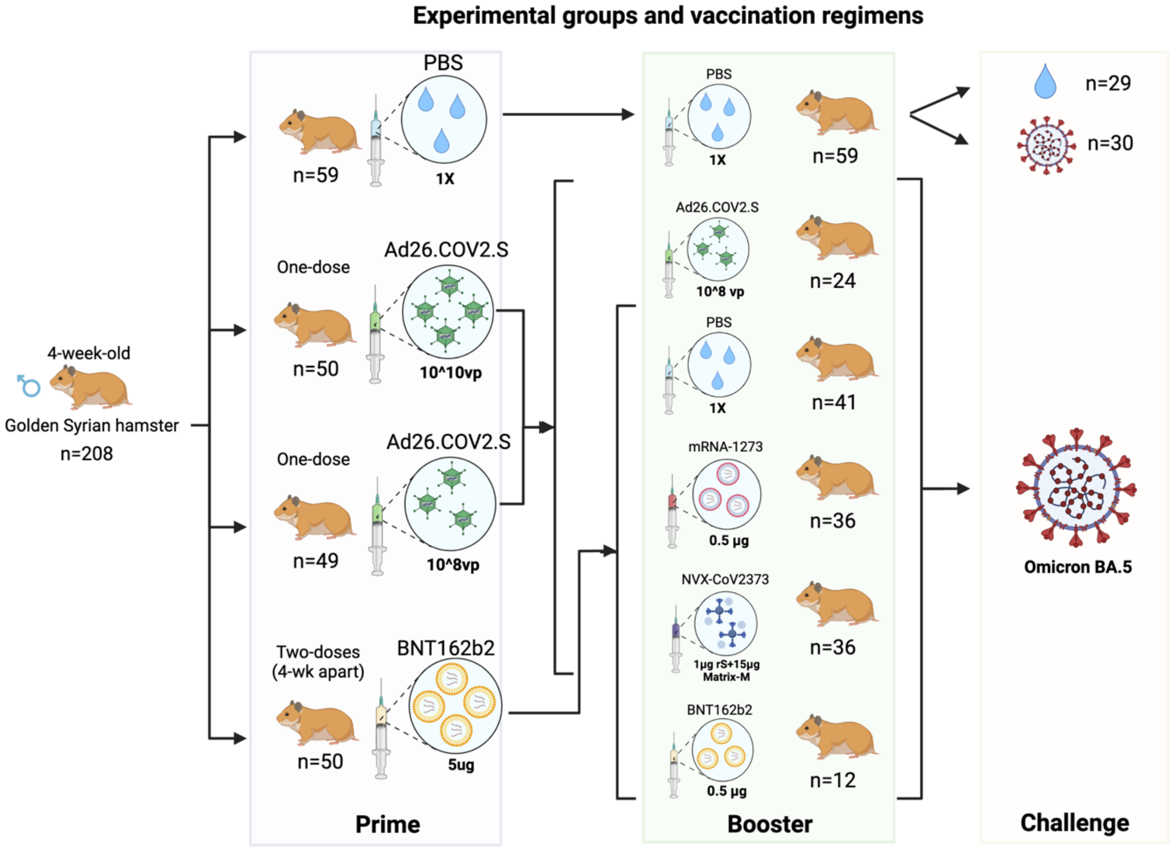

Supplementary Fig. 2. Schematic representation of vaccination regimens used in the study. One group of 50 and one of 49, 4-5 week-old male Golden Syrian hamsters (HsdHan: AURA strain, Envigo Indianapolis, IN), were vaccinated with 1 × 10^8 and 1 x 10^{10} viral particles of Ad26.COV2.S vaccines, respectively. Vaccines were delivered intramuscularly in one 100 µL dose in the hind leg. Another group of 50 were immunized three weeks apart with 5µg of BNT162b2 vaccine (100 µL/dose). Additionally, a placebo (PBS 1X) group of 59 animals were included. All three of the groups vaccinated with 10^{10} vp Ad26.COV2.S, 10^8 vp Ad26.COV2.S or 5µg of BNT162b2 vaccines were heterologous boosted with the same vaccines: mRNA-1273 (0.5 µg) and NVX-CoV2373 (1µg rS/15µg Matrix-M). A homologous booster was also used, 10^8 vp Ad26.COV2.S for both groups primary vaccinated with Ad26.COV2.S vaccine and 0.5 µg of BNT162b2 for the group primed with this vaccine. In addition, a PBS boosted group was included for comparison. Animals were challenged with 10^4 PFU/dose of Omicron BA.5 or PBS (mock group). The Fig. was created using BioRender (biorender.com).

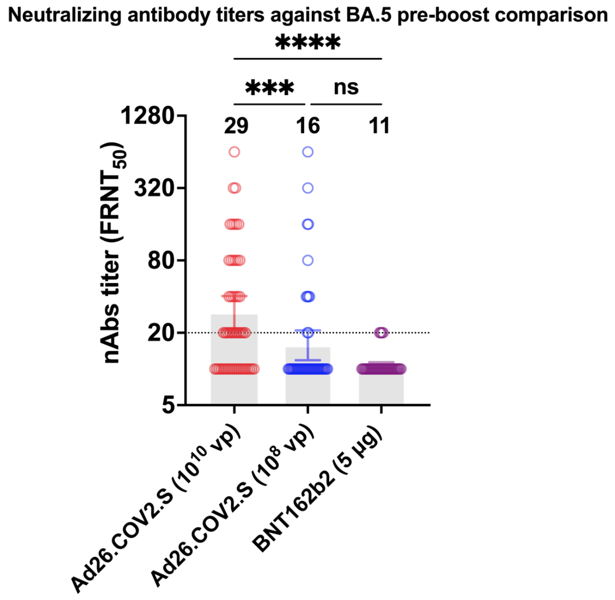

Supplementary Fig. 3. Neutralizing antibody titers against BA.5 pre-boost comparison between hamsters primed with Ad26.COV2.S (10^{10} or 10^{8} vp) or BNT162b2 vaccines. 4-week-old male Golden Syrian hamster were immunized either with one dose 10^{10} or 10^{8} vp of Ad26.COV2.S or two doses of BNT162b2 (5 µg/each) vaccines. After 6 months (day 168) of primary vaccination, animals were bled for antibody analysis. Serum neutralizing antibody responses pre-boost against BA.5 in hamsters primary vaccinated with 10^{10} vp Ad26.COV2.S (right, n=50), 10^{8} vp Ad26.COV2.S (center, n=49) or 5 µg BNT162b2 (left, n=50). Each data point represents the 50% SARS-CoV-2-neutralizing titer ($FRNT_{50}$) of a serum sample, bars illustrate GMTs, the whisker bars represent the 95% CI, GMTs are indicated at the top of the graphs with dotted lines representing the LOD. Kruskal-Wallis test with Dunn's multiple comparison post-test (ns, not significant, $p = 0.3005$; ***$p = 0.0002$; ****$p < 0.0001$). See Source Data for complete data.

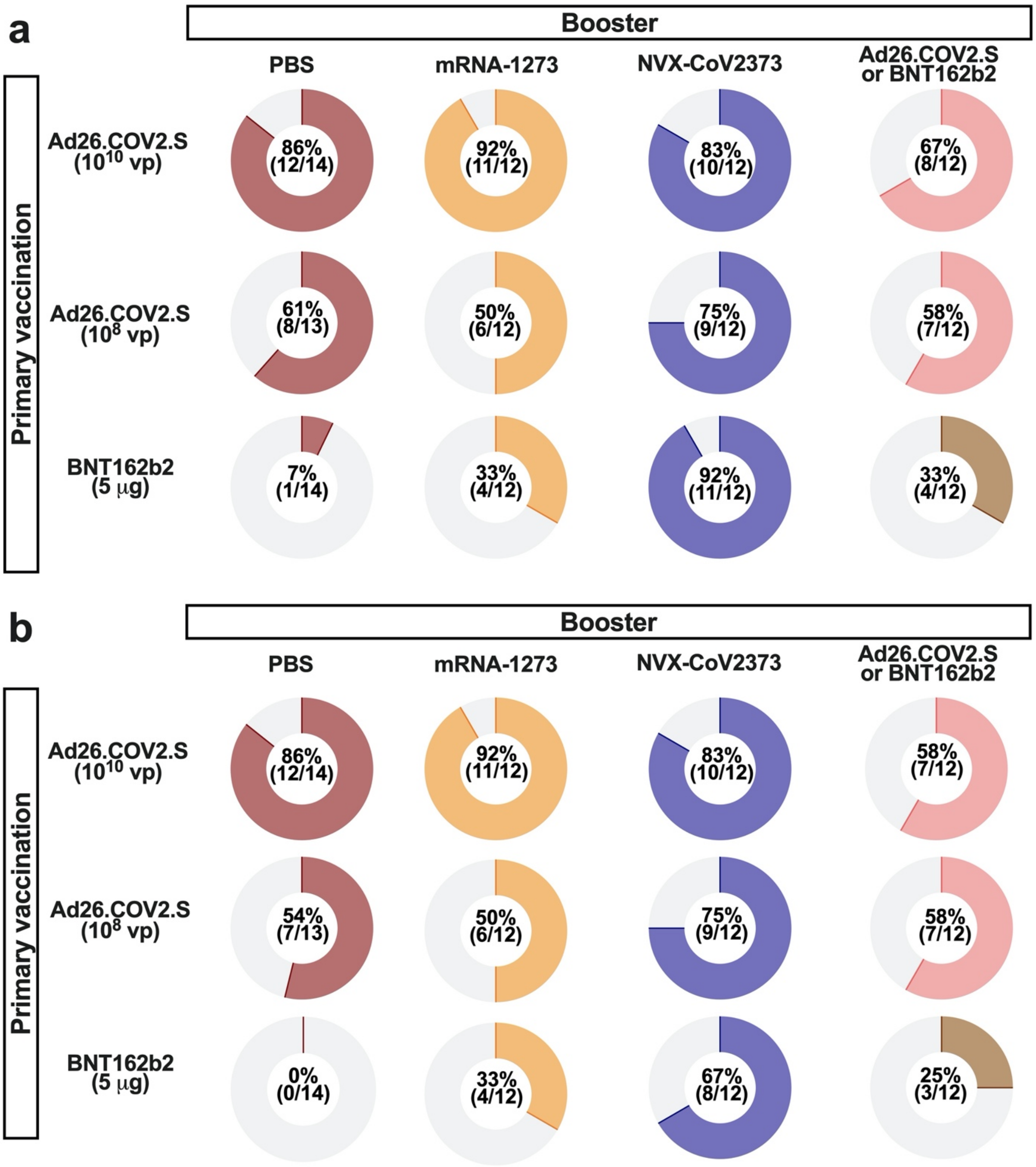

Supplementary Fig. 4. Proportion of hamsters seroconverted, 1mo post- and 3mo post-boost against Omicron BA.5 sublineage. Proportion of hamsters seroconverted **(a)** 1 month post-boost and **(b)** 3 months post-boost. Percentage (%) and number of animals with detectable nAbs against BA.5 ($FRNT_{50}$≥20, LOD=20) are shown inside each graph. Colors designate experimental cohorts (n= 12, 13 or 14 animals per group as indicated). The color-coded portion of the ring represents the proportion of hamsters seroconverted against Omicron BA.5. Panels were generated using GraphPad Prism v9.4 software. See Supplementary Table 1 for complete data.

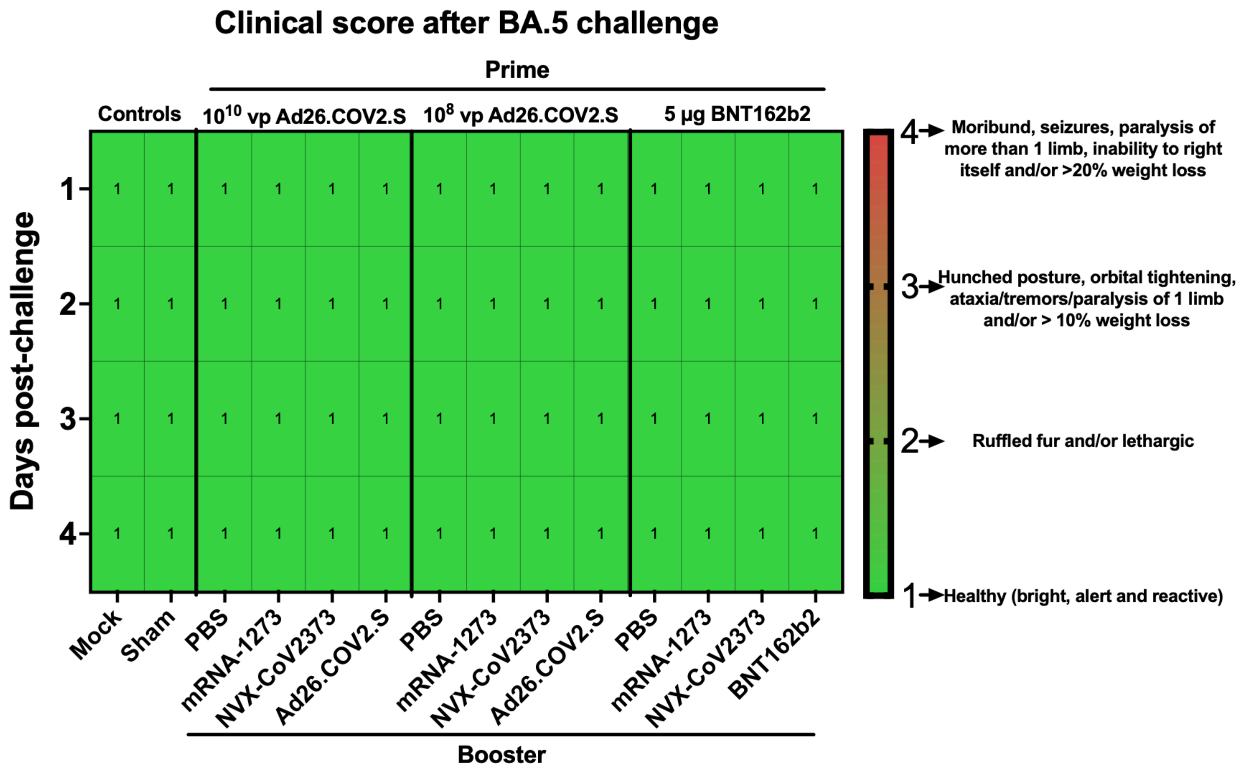

Supplementary Fig. 5. Heat map of clinical score after Omicron BA.5 challenge. All hamsters were healthy (bright, alert and reactive – score 1) up to 4 days post-challenge (dpc). Even though the sham vaccinated group lost more weight in comparison with the other groups, no difference in the clinical signs of disease were observed until 4 dpc. Scoring was based on the following criteria, 1= Healthy (bright, alert and reactive); 2 = Ruffled fur and/or lethargic; 3 = A score of 2 plus 1 additional clinical sign such as, hunched posture, orbital tightening, or neurological signs such as ataxia/tremors/paralysis of 1 limb, and/or > 10% weight loss; 4= A score of 3 plus 1 additional clinical sign such as, reluctance to move when stimulated, or neurologic signs (seizures, paralysis of more than 1 limb, inability to right itself) or >20% weight loss. The numbers within each quadrant represent the mean value of the clinical score for each group of animals. See Source Data for the complete daily monitoring of each animal after challenge with Omicron BA.5.

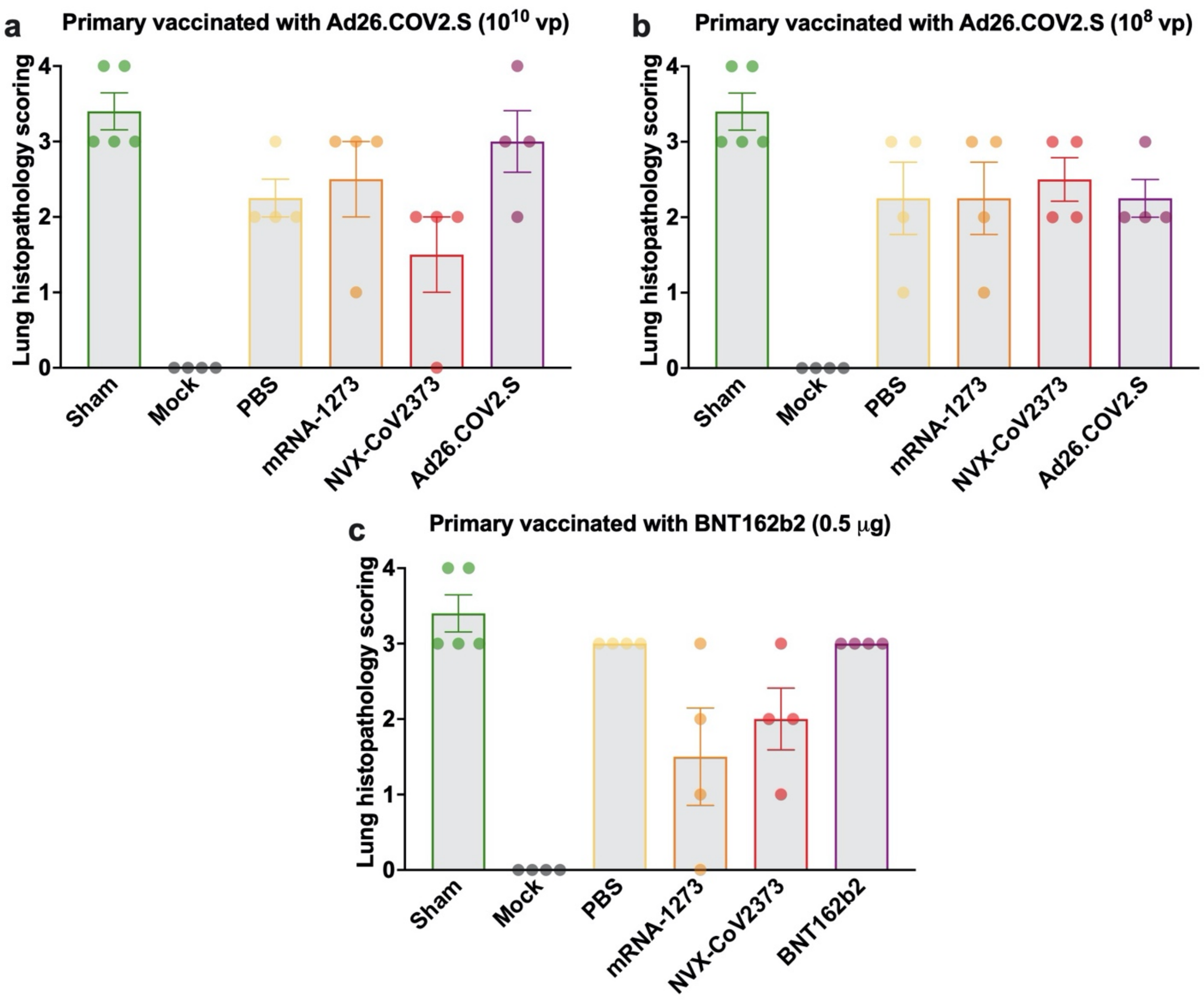

Supplementary Fig. 6. Histopathological severity scores of lungs of vaccinated and boosted Syrian hamsters challenged with Omicron subvariant BA.5. Histopathology scoring of the left lung of hamsters primary vaccinated with **(a)** one dose 10^{10} vp or **(b)** 10^{8} vp of Ad26.COV2.S or **(c)** two doses of BNT162b2 (0.5 µg per dose) vaccines, four days after intranasal challenge with 10^{4} PFU of BA.5. To evaluate comprehensive histological changes, lung tissue sections were scored based on pathological changes by using the following scoring system: 0 (none, 0%), 1 (minimal, 1%–25%), 2 (mild, 26%–50%), 3 (moderate,51%–75%), or 4 (severe,76%–100%). The total score is shown for individual animals. Data are presented as means ± SEM. See Source Data for the complete histopathological evaluation of each specimen.

Correlation between neutralizing antibody titers and protection against BA.5 at 2 dpi

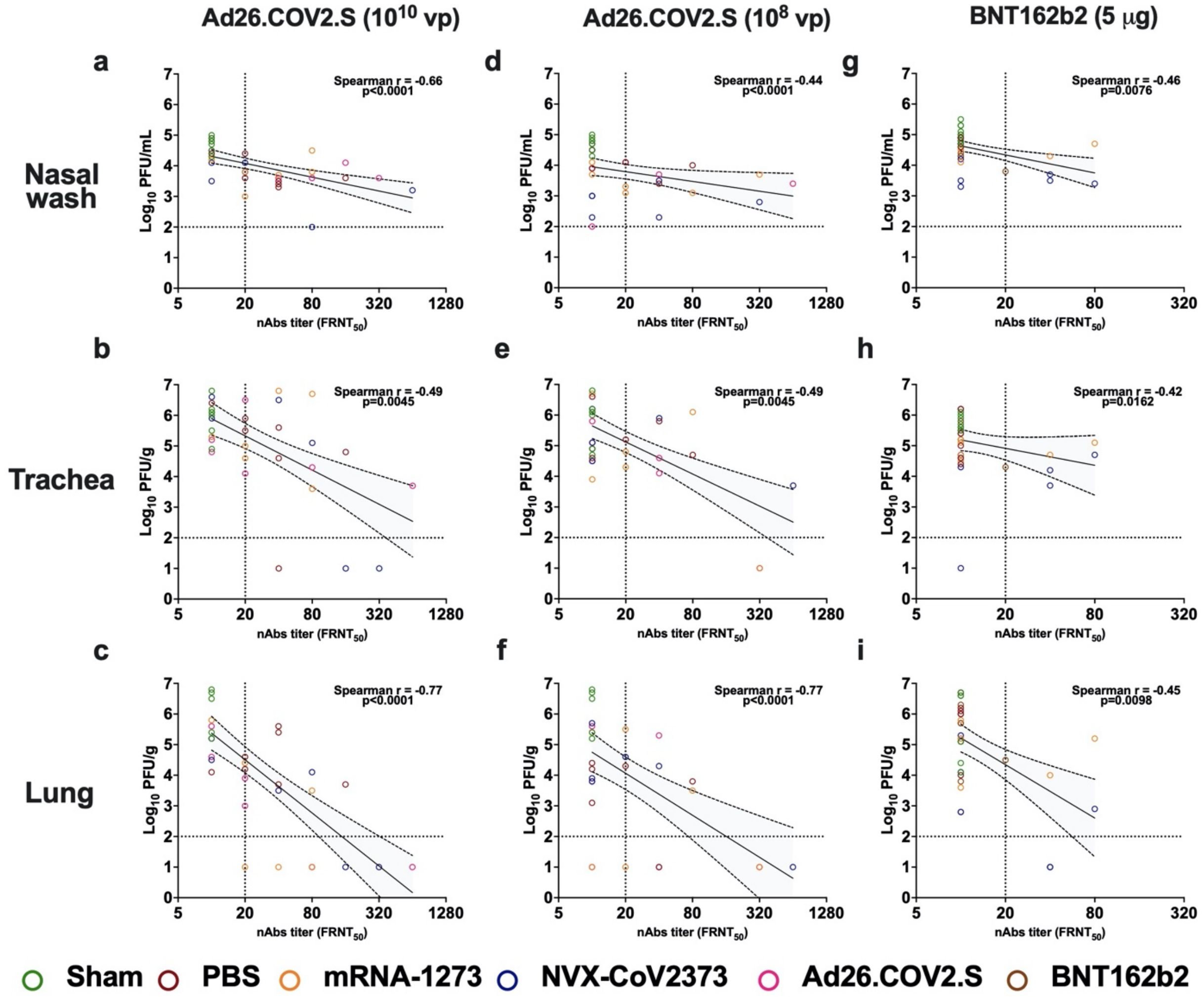

Supplementary Fig. 7. Correlation between neutralizing antibody titers and protection against BA.5 at 2 dpi. a-i Correlation between the $FRNT_{50}$ neutralization titer at day 252 post-vaccination (pre-challenge) against BA.5 (x-axis) and the infectious virus titer (y-axis) detected 2 dpi in nasal washes (**a**, **d** and **g**), trachea (**b**, **e** and **h**) and lungs (**c**, **f** and **i**) of hamsters primed with one dose 10^{10} vp of Ad26.COV2.S (**a**, **b** and **c**), one dose 10^{8} vp of Ad26.COV2.S (**d**, **e** and **f**) or two doses 5 μg of BNT162b2 vaccines (**g**, **h** and **i**). The experimental groups are color coded (n = 6-8 animals per group), dots illustrate each specimen, dotted lines show LOD; Linear regression (thick lines), 95% CI (dashed lines and shading), Spearman's rank correlation coefficient (r) and p values are shown. See Source Data for complete data.

Supplementary Table 1. Geometric mean titers (GMTs) and 95%CI of neutralizing antibody titers and fraction plus percentage (%) of animals seroconverted, pre-, 1mo post- and 3mo post-boost against Omicron BA.5 sublineage. mo, month(s); dpv, days post-vaccination; vp, viral particles; µg, micrograms; $FRNT_{50}$, 50% focus reduction neutralization antibody titer; N/A, not applicable.

Prime	Booster	Number of hamsters per time-point	Neutralizing antibodies (FRNT50) against BA.5 - Geometric mean titer (GMT) and 95%CI			Fraction and percentage (%) of animals seroconverted		
			Days post-vaccination (dpv)			Days post-vaccination (dpv)		
			168 (pre-boost)	196 (1mo post-boost)	251 (pre-challenge)	168 (pre-boost)	196 (1mo post-boost)	251 (pre-challenge)
PBS	PBS	59	10 (10-10)	10 (10-10)	10 (10-10)	N/A	N/A	N/A
Ad26.COV2.S (10^10vp)	PBS	14	36 (22-61)	33 (19-56)	31 (18-53)	12/14 (85.7)	12/14 (85.7)	12/14 (85.7)
Ad26.COV2.S (10^10vp)	mRNA-1273 (0.5µg)	12	28 (13-59)	42 (24-75)	40 (22-72)	8/12 (66.7)	11/12 (91.7)	11/12 (91.7)
Ad26.COV2.S (10^10vp)	NVX-CoV2373 (1µg rS/15ug Matrix-M)	12	30 (12-73)	80 (33-193)	63 (23-172)	6/12 (50.0)	10/12 (83.3)	10/12 (83.3)
Ad26.COV2.S (10^10vp)	Ad26.COV2.S (10^8vp)	12	25 (13-49)	30 (14-63)	25 (13-50)	8/12 (66.7)	8/12 (66.7)	7/12 (58.3)
Ad26.COV2.S (10^8vp)	PBS	13	25 (13-47)	26 (14-50)	22 (12-40)	8/13 (61.5)	8/13 (61.5)	7/13 (53.8)
Ad26.COV2.S (10^8vp)	mRNA-1273 (0.5µg)	12	15 (8-28)	25 (11-57)	25 (11-55)	3/12 (25.0)	6/12 (50.0)	6/12 (50.0)
Ad26.COV2.S (10^8vp)	NVX-CoV2373 (1µg rS/15ug Matrix-M)	12	13 (9-15)	50 (23-110)	50 (22-115)	2/12 (16.7)	9/12 (75.0)	9/12 (75.0)
Ad26.COV2.S (10^8vp)	Ad26.COV2.S (10^8vp)	12	20 (8-48)	30 (12-74)	28 (12-69)	3/12 (25.0)	7/12 (58.3)	7/12 (58.3)
Bnt162b2 (5µg)	PBS	14	10 (10-10)	10 (9-12)	10 (10-10)	0/14 (0.0)	1/14 (7.1)	0/14 (0.0)
Bnt162b2 (5µg)	mRNA-1273 (0.5µg)	12	11 (9-13)	16 (10-26)	15 (9-24)	2/12 (16.7)	4/12 (33.3)	4/12 (33.3)
Bnt162b2 (5µg)	NVX-CoV2373 (1µg rS/15ug Matrix-M)	12	11 (9-12)	32 (17-58)	34 (17-66)	1/12 (8.3)	11/12 (91.7)	8/12 (66.7)
Bnt162b2 (5µg)	Bnt162b2 (0.5µg)	12	11 (9-13)	15 (10-22)	11 (9-13)	1/12 (8.3)	4/12 (33.3)	3/12 (25.0)

Supplementary Table 2. Fold change reduction of infectious virus titer (virus load) in nasal washes, trachea and lung 2- and 4-days post-infection (dpi) with Omicron BA.5. The fold change reduction was calculated as the ratio of the mean values. Sham-vaccinated group was used for comparison and fold change reduction calculation. Comma (,) were used as thousands separator. Numbers are color scaled for greater visualization from the lowest to the highest values (green ⇢ white ⇢ red). For a comparable data visualization, the color scale was applied separately in each specimen at each time-point from each primary vaccinated group.

Specimen	Days post-infection (dpi)	Primary vaccinated with Ad26.COV2.S (10^10 vp)				Primary vaccinated with Ad26.COV2.S (10^8 vp)				Primary vaccinated with BNT162b2 (5 µg)			
		PBS (1X)	mRNA-1273 (0.5µg)	NVX-CoV2373 (1µg)	Ad26.COV2.S (10^8vp)	PBS (1X)	mRNA-1273 (0.5µg)	NVX-CoV2373 (1µg)	Ad26.COV2.S (10^8vp)	PBS (1X)	mRNA-1273 (0.5µg)	NVX-CoV2373 (1µg)	BNT162b2 (0.5 µg)
Nasal Wash	2 dpi	6	5	9	2	3	13	56	6	2	4	27	3
Trachea		2	1	3	1	2	1	12	0	12	69	262	25
Lung		53	53	77	16	47	54	57	52	3	20	97	7
Nasal Wash	4 dpi	4	3	32	2	32	21	21	2	3	5	1,012	15
Trachea		1	14	86,257	1	1	7	259	1	0	0	16,339	2
Lung		289	686,888	686,888	50	242	55	140	30	19	122	3,059	58

Supplementary Table 3. Analysis of agreement between neutralizing titer (FRNT$_{50}$) and infectious virus titer in lungs at 4 days post-infection. Sensitivity, specificity, positive and negative predictive values (PPV and NPV, respectively) and Cohen's Kappa coefficient (value of kappa) are shown, with 95% confidence interval (CI).

	**Infectious virus titer ≥ 100 PFU/g**	**Infectious virus titer < 100 PFU/g**	**Total**	**Sensitivity (95% CI)**	**Specificity (95% CI)**	**Positive predictive value - PPV (95% CI)**	**Negative predictive value - NPV (95% CI)**	**Cohen's Kappa coefficient (95% CI)**
**FRNT$_{50}$ ≥ 20**	40	4	44	97.6% (86-100%)	92% (81-97%)	90.9% (80-96%)	97.9% (87-100%)	0.89 (0.79-0.98)
**FRNT$_{50}$ < 20**	1	46	47					
**Total**	41	50	91					
