# Supplementary material for: Immunogenicity and efficacy of vaccine boosters against SARS-CoV-2 Omicron subvariant BA.5 in male Syrian hamsters
Source: Nat Commun. 2023 Jul 17;14:4260. doi: 10.1038/s41467-023-40033-2 (PMC10352277; doi:10.1038/s41467-023-40033-2)
Supplement: Supplementary file 3 — Reporting Summary [file 41467_2023_40033_MOESM3_ESM.pdf]

nature portfolio

Corresponding author(s): Kenneth S. Plante and Scott C. Weaver

Last updated by author(s): Jun 7, 2023

Reporting Summary

Nature Portfolio wishes to improve the reproducibility of the work that we publish. This form provides structure for consistency and transparency in reporting. For further information on Nature Portfolio policies, see our Editorial Policies and the Editorial Policy Checklist.

Statistics

For all statistical analyses, confirm that the following items are present in the figure legend, table legend, main text, or Methods section.

n/a	Confirmed	
☐	☒	The exact sample size (*n*) for each experimental group/condition, given as a discrete number and unit of measurement
☐	☒	A statement on whether measurements were taken from distinct samples or whether the same sample was measured repeatedly
☐	☒	The statistical test(s) used AND whether they are one- or two-sided<br>*Only common tests should be described solely by name; describe more complex techniques in the Methods section.*
☐	☒	A description of all covariates tested
☐	☒	A description of any assumptions or corrections, such as tests of normality and adjustment for multiple comparisons
☐	☒	A full description of the statistical parameters including central tendency (e.g. means) or other basic estimates (e.g. regression coefficient) AND variation (e.g. standard deviation) or associated estimates of uncertainty (e.g. confidence intervals)
☐	☒	For null hypothesis testing, the test statistic (e.g. *F*, *t*, *r*) with confidence intervals, effect sizes, degrees of freedom and *P* value noted<br>*Give P values as exact values whenever suitable.*
☒	☐	For Bayesian analysis, information on the choice of priors and Markov chain Monte Carlo settings
☒	☐	For hierarchical and complex designs, identification of the appropriate level for tests and full reporting of outcomes
☒	☐	Estimates of effect sizes (e.g. Cohen's *d*, Pearson's *r*), indicating how they were calculated

Our web collection on statistics for biologists contains articles on many of the points above.

Software and code

Policy information about availability of computer code

Data collection	Cytation7 Imagining Reader (model #CYT7U, BioTek) and VersaMax Microplate Reader (Molecular Devices, CA, USA)
Data analysis	Hematoxylin and eosin (H&E) images were analyzed using Olympus cellSens Entry (version 3.2 software). Data analysis and graphing were performed in Prism (version 9.5.1, GraphPad).

For manuscripts utilizing custom algorithms or software that are central to the research but not yet described in published literature, software must be made available to editors and reviewers. We strongly encourage code deposition in a community repository (e.g. GitHub). See the Nature Portfolio guidelines for submitting code & software for further information.

Data

Policy information about availability of data

All manuscripts must include a data availability statement. This statement should provide the following information, where applicable:

- Accession codes, unique identifiers, or web links for publicly available datasets
- A description of any restrictions on data availability
- For clinical datasets or third party data, please ensure that the statement adheres to our policy

Source data are provided with this paper. All data supporting the findings in this study are also available from the corresponding authors upon request.

Human research participants

Policy information about studies involving human research participants and Sex and Gender in Research.

Reporting on sex and gender	N/A
Population characteristics	N/A
Recruitment	N/A
Ethics oversight	N/A

Note that full information on the approval of the study protocol must also be provided in the manuscript.

Field-specific reporting

Please select the one below that is the best fit for your research. If you are not sure, read the appropriate sections before making your selection.

☒ Life sciences ☐ Behavioural & social sciences ☐ Ecological, evolutionary & environmental sciences

For a reference copy of the document with all sections, see nature.com/documents/nr-reporting-summary-flat.pdf

Life sciences study design

All studies must disclose on these points even when the disclosure is negative.

Sample size	Sample size was determined based on previous experience measuring antibody response and viral loads in tissues comparing vaccinated and controls animals infected with SARS-CoV-2, by the NIH-SAVE network (https://www.niaid.nih.gov/research/sars-cov-2-assessment-viral-evolution-program and "Defining the risk of SARS-CoV-2 variants on immune protection" - DOI:10.1038/s41586-022-04690-5). A minimum of six animals per group and per timepoint was used.
Data exclusions	No data were excluded from analysis.
Replication	Animal experiments were done once according to the number of subjects indicated by power analyses, which were the maximum number of animals approved by the UTMB IACUC in accordance with the principle of reduction. Because of the high number of samples, viral loads and neutralization assays were performed once with variance determined by among-animal data, also using several internal controls to assure reproducibility.
Randomization	Hamsters were received from Envigo by dedicated animal research personnel at UTMB, who randomly assigned the hamsters to cages of 3 or 4 animals with no additional knowledge of study design. No further randomization was performed by research personnel.
Blinding	Blinding was not possible during animal manipulations due to safety considerations regarding vaccinated and infected animals. Blinding was done for the pathologist reviewing the lung histology and for the antibody assays.

Reporting for specific materials, systems and methods

We require information from authors about some types of materials, experimental systems and methods used in many studies. Here, indicate whether each material, system or method listed is relevant to your study. If you are not sure if a list item applies to your research, read the appropriate section before selecting a response.

Materials & experimental systems

n/a	Involved in the study	
☐	☒	Antibodies
☐	☒	Eukaryotic cell lines
☒	☐	Palaeontology and archaeology
☐	☒	Animals and other organisms
☒	☐	Clinical data
☒	☐	Dual use research of concern

Methods

n/a	Involved in the study	
☒	☐	ChIP-seq
☒	☐	Flow cytometry
☒	☐	MRI-based neuroimaging

March 2021

Antibodies

Antibodies used	FRNTs used rabbit polyclonal antibodies against SARS-CoV nucleocapsid (Shinji Makino, UTMB, non-commercial). HRP-conjugated goat anti-rabbit antibody (Cell Signaling Technology, cat 7074, lot 28) was used for detection. ELISAs used HRP-conjugated goat anti-hamster IgG (Thermo Fisher Scientific, cat PA1-29626), as secondary antibody.
Validation	HRP-conjugated antibodies were tested by the manufacturers to demonstrate its ability to be used as secondary antibody. Privately provided antibodies were not independently validated, but previously used as described on Vanderheiden, A. et al. Development of a Rapid Focus Reduction Neutralization Test Assay for Measuring SARS-CoV-2 Neutralizing Antibodies. Curr. Protoc. Immunol. 131, 116 (2020) and Plante, J. A. et al. Vaccination Decreases the Infectious Viral Load of Delta Variant SARS-CoV-2 in Asymptomatic Patients. Viruses 14, 2071 (2022).

Eukaryotic cell lines

Policy information about cell lines and Sex and Gender in Research

Cell line source(s)	VeroE6 cell line expressing human TMPRSS2 were obtained from JCRB Cell Bank (JCRB1819, lot 04172020).
Authentication	None of the cell lines were authenticated.
Mycoplasma contamination	All cell lines tested negative for mycoplasma.
Commonly misidentified lines (See ICLAC register)	No commonly misidentified cell lines were used in this study.

Animals and other research organisms

Policy information about studies involving animals; ARRIVE guidelines recommended for reporting animal research, and Sex and Gender in Research

Laboratory animals	This study utilized 4-to-5-week-old male golden Syrian hamsters (strain HsdHan:AURA) obtained from Envigo.
Wild animals	Wild animals were not used in the study.
Reporting on sex	The findings apply only for male hamsters. The sex of animals were not considered in the study design.
Field-collected samples	Field-collected samples were not used in this study.
Ethics oversight	The study protocol was approved by the Institutional Animal Care and Use Committee (IACUC) at the University of Texas Medical Branch (UTMB).

Note that full information on the approval of the study protocol must also be provided in the manuscript.
